# Dual Cross-Attention Network for Hierarchical Feature Fusion in Protein-Protein Interaction Prediction

**DOI:** 10.34133/csbj.0200

**Published:** 2026-08-17

**Authors:** Shuai Lu, Yuguang Li, Zhen Tian, Xiaofei Nan, Qinglei Zhou, Shoutao Zhang

**Affiliations:** ^1^ School of Computer and Artificial Intelligence, Zhengzhou University, Zhengzhou 450001, Henan, China.; ^2^ National Supercomputing Center in Zhengzhou, Zhengzhou University, Zhengzhou 450001, Henan, China.; ^3^ School of Life Sciences, Zhengzhou University, Zhengzhou 450001, Henan, China.; ^4^ Zhongyuan Intelligent Medical Laboratory, School of Life Sciences, Henan Institute of Science and Technology, Xinxiang 453000, Henan, China.

## Abstract

Characterizing protein-protein interactions (PPIs) is essential for deciphering core biological processes, including signal transduction, metabolic pathway regulation, immune recognition, and cell cycle control. However, experimental PPI determination remains time-consuming and expensive, driving the adoption of deep learning as an efficient and accurate computational approach. Current deep-learning-based PPI prediction models typically process both intra- and inter-protein as isolated units in feature extraction, thereby ignoring mutual information transfer within a single protein and the interacting pair. To address this limitation, we propose DCAPPI (Dual Cross-Attention network for Protein-Protein Interaction prediction), a novel framework leveraging dual cross-attention modules for hierarchical feature fusion at both intra- and inter-protein levels. First, the Channel Cross-Attention module processes protein sequence and structure as distinct input channels. It generates deep intra-protein representations by performing cross-attention between sequence-derived and structure-derived tokens, achieving multimodal feature integration. Second, the Partner Cross-Attention module models the target protein and its interacting partner as a pair of correlative units. By performing cross-attention operations across these units, it enables collaborative feature fusion and constructs context-aware inter-protein interaction features. Evaluation results indicate that DCAPPI achieves superior performance over state-of-the-art methods on benchmark datasets.

## Introduction

Protein-protein interactions (PPIs) are fundamental to cellular processes such as signal transduction, metabolic regulation, and disease pathogenesis [1,2]. Accurate PPI prediction enables deeper insights into exploring molecular mechanisms and biological functions and identifying therapeutic targets [3]. However, traditional experimental methods like yeast 2-hybrid screening, tandem affinity purification, mass spectrometry, proteome chips, and microarray technology are costly and time-consuming [4,5]. Furthermore, inherent experimental variability, operational errors, and other subjective or objective factors often introduce deviations between experimental results and actual biological interactions, potentially generating a large amount of false-positive or false-negative data [6]. These limitations highlight that experimental methods alone are insufficient to generate comprehensive, high-quality, and reliable PPI data in the postgenomic era [7].

To overcome these challenges, computational methods for predicting PPI have emerged as essential complementary tools. Early computational efforts relied on molecular dynamics simulations [8] and docking [9] to model protein complex binding in simulated environments [10]. These methods are computationally intensive. Their requirement for high-precision protein structures limits large-scale applications, making them impractical for high-throughput binary PPI prediction [11].

The development of high-throughput sequencing has exponentially expanded protein sequence databases, driving the development of machine learning (ML) methods for PPI prediction. These methods leveraging support vector machines [12–14], naive Bayes [15], random forests [16,17], and *k*-nearest neighbors [18] became prevalent, each usually requiring specialized feature sets. These models often utilize sequence-derived features, including multiscale local descriptors capturing interaction information from continuous amino acid segments [13], and physicochemical properties combined with fuzzy membership functions improving classical algorithms like support vector machines [19]. Although these ML methods no longer strictly rely on experimentally solved structures for prediction, they still require a large amount of expert-driven feature engineering and domain knowledge [11].

Driven by the rapid increase in protein sequence data, deep learning (DL) techniques such as deep neural networks (DNNs) [20], convolutional neural networks (CNNs) [21], recurrent neural networks [22], and autoregressive models [23], have revolutionized PPI prediction. They automate feature extraction from large-scale datasets, reducing the need for expert-driven feature engineering [24]. These methods effectively capture complex patterns and nonlinear dependencies in protein sequences. For example, DeepPPI [25] shows superior performance over ML-based methods using stacked fully connected layers. DNN-PPI [26] treats sequences as natural language, using CNNs and long short-term memory networks to capture local features. PIPR [27] incorporates residual recurrent CNNs in Siamese architectures to leverage contextualized information. EnsDNN [28] utilizes an ensemble DNN trained on various sequence descriptors such as auto covariance, local descriptor, and multiscale local descriptor. DPPI [29] combines Siamese-like CNNs with random projection and augmentation using evolutionary information. DeepSG2PPI [30] fused 2-dimensional (2D) CNNs for local context with 1-dimensional (1D) CNNs for global statistics and biological features. MARPPI [31] utilized multiscale residual networks with Res2vec. KSGPPI [32] integrated ESM-2 [33] embeddings with *k*-spaced amino acid pairs via 2D CNNs and graph embeddings. Despite their success, sequence-based methods (both ML and DL) inherently struggle to model long-range dependencies and accurately identify neighboring residues in 3-dimensional (3D) chemical space, which is critical for identifying interaction sites [11].

Proteins perform functions through 3D conformations and engage in interactions within the structural space, which has led to the development of structure-based prediction methods [34,35]. For instance, PrePPI [36] combines 3D structural information with functional clues using naive Bayes. Struct2Graph [37] employs weight-shared graph convolutional networks and mutual attention on structural data fed into a feedforward network. HIGH-PPI [38] utilizes hierarchical graph neural networks (GNNs) leveraging 3D structures and existing PPI relationships. Beyond dimer PPI, PLO3S [39] demonstrates a coarse-to-fine screening hierarchy on protein surfaces, positioning itself as a prefilter before finer methods. Meanwhile, Lu *et al.* [40] surveyed multimer prediction challenges and noted that even state-of-the-art dimer hybrids lack clear paths. A major obstacle for structure-based methods remains the limited availability of experimentally determined high-resolution 3D structures.

The breakthrough success of protein structure prediction [41–44] and protein language models (PLMs) [33,45] in accurately predicting and embedding protein 3D structures opens new avenues for many bioinformatics tasks [46–49]. This enables hybrid methods leveraging both semantic features from sequences and spatial features from predicted structural information to enhance PPI prediction. For instance, TAGPPI [50] calculates residue contact maps (2D structures) from AlphaFold v2 (AF2)-predicted structures, uses SeqVec [51] for residue embeddings, and applies stacked graph attention networks (GATs) [52] to protein pairs, outperforming sequence-only methods. SGPPI [53] refines this by filtering residues with low relative solvent accessibility (<0.2) to focus on those more relevant to interaction. TAGPPI [50] utilizes CNNs for sequence features and GATs [52] on contact maps generated from AF2-predicted structures, embedding residues by SeqVec and fusing those features for prediction. Recently, CollaPPI [11] integrates protein-level collaboration via mutual attention networks and task-level collaboration through homogeneous multitask learning in a Siamese architecture. PPI-BAN [7] applies 1D CNNs to extract sequence features from proteins and GearNet [54] to learn 3D structural features while employing a deep bilinear attention network to model joint features between sequence and structure explicitly. CollaPPI and PPI-BAN extract residue feature embedding by running a pre-trained PLM ESM-2 [33]. Despite enhanced performance, those hybrid methods use oversimplified sequence–structure fusion strategies. Typical techniques include direct feature concatenation or aggregating sequence features as node attributes in GNN-based structural frameworks. Moreover, these approaches underutilize critical information governing how the protein interacts with its partner. Specifically, existing methods treat the input protein pair as separate entities and rely on basic operations such as matrix concatenation or element-wise multiplication. This strategy of protein–partner feature fusion cannot fully capture interaction patterns and share knowledge between them. The recent compound–protein interaction prediction method FilmCPI also shows that letting the richer-modality side modulate the scarcer-modality side yields better than symmetric fusion [47]. Additionally, Struc2Graph [37] and CollaPPI [11] utilize mutual attention to construct collaborative features, allowing for the sharing of cross-protein information while compressing feature dimensions.

To overcome these limitations, we propose a novel PPI prediction network named DCAPPI (Dual Cross-Attention network for Protein-Protein Interaction prediction). Our framework utilizes a dual cross-attention network for hierarchical feature fusion across sequence–structure and protein–partner from the input protein pairs. DCAPPI employs a dual cross-attention network consisting of the Channel Cross-Attention (CCA) module and the Partner Cross-Attention (PCA) module. DCAPPI operates through 3 integrated components, namely, the CCA module, PCA module, and a multitask collaboration (MTC) module. Firstly, the CCA module extracts sequence features using bidirectional gated recurrent units (BiGRUs) and constructs structural embeddings via GATs and then integrates sequence and structure channel features and performs intra-protein fusion of these channel-specific representations through cross-attention operation. Then, the PCA module processes protein pairs by cross-attention and mutual attention operations to integrate protein–partner features, enabling inter-protein fusion and dimensional transformation while capturing interaction contexts. Finally, inspired by CollaPPI [11], task-level collaboration is implemented through homogeneous multitask learning that incorporates function prediction and subcellular localization, facilitating knowledge transfer via auxiliary biological tasks. Experimental results on benchmark datasets confirm that DCAPPI outperforms state-of-the-art approaches in PPI prediction. There are 4 innovations in our work:

• We propose DCAPPI, which is a novel PPI prediction framework integrating dual cross-attention modules (CCA and PCA modules) for hierarchical feature fusion at intra- and inter-protein levels, addressing the limitation of isolated feature processing in existing methods.
• The CCA module enables bidirectional information transfer between sequence context representations and spatial topology representations via standard multihead cross-attention operation, achieving more comprehensive intra-protein representation than simple concatenation.
• The PCA module constructs context-aware inter-protein interaction features through standard multihead cross-attention and mutual attention operations, dynamically capturing binding units and coevolutionary signals between the target protein and its partner.
• DCAPPI outperforms state-of-the-art methods on 3 benchmark datasets (the Yeast, Multi-species, and Multi-class datasets), demonstrating superior predictive accuracy, strong generalization under low sequence identity, and robustness in classifying diverse interaction types.

We emphasize that the attention operators in CCA and PCA modules are standard multihead cross-attention. Our contribution is the task-specific architectural design that organizes these operators into a biologically motivated hierarchical fusion pipeline tailored for PPI prediction.

## Methods

**Fig. 1.**
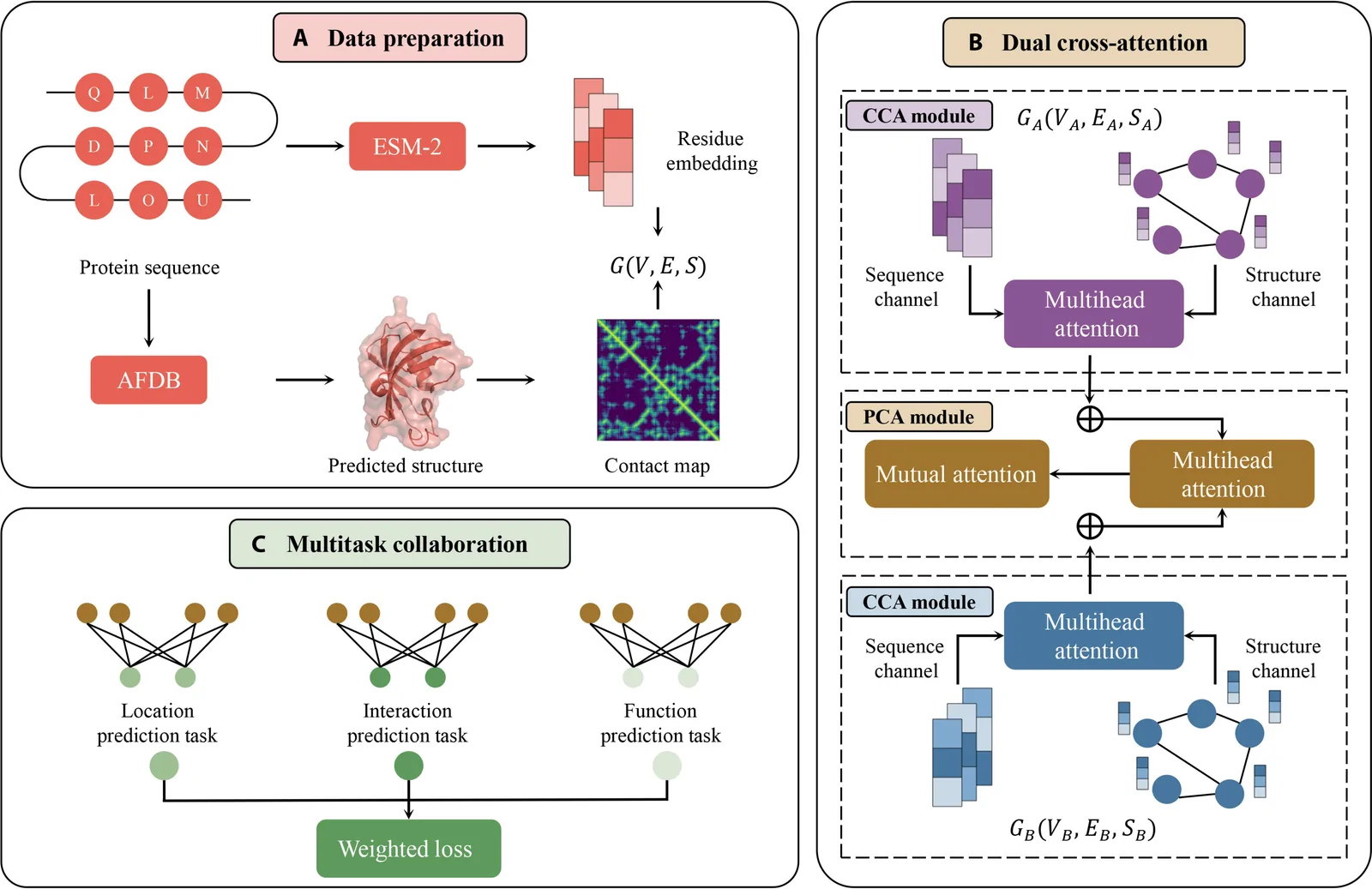
The workflow of DCAPPI (Dual Cross-Attention network for Protein-Protein Interaction prediction). (A) DCAPPI employs the pre-trained protein language model ESM-2 to generate sequence embedding features and uses the predicted 3-dimensional (3D) structures from the AlphaFold Protein Structure Database (AFDB) to construct residue-level structural graphs. (B) The Channel Cross-Attention (CCA) module separately fuses the sequence embeddings and structural graphs for each protein in the input pair (proteins *A* and *B*). The Partner Cross-Attention (PCA) module then captures the interaction context between the 2 proteins and conducts mutual attention to achieve dimension alignment. (C) We leverage collaborative knowledge from 2 additional protein-related classification tasks, namely, protein function prediction and subcellular localization prediction, to further boost the performance of protein-protein interaction classification.

As shown in Fig. 1, we employ a dual cross-attention framework in DCAPPI, integrating sequential and structural protein representations for PPI while extracting interacting relationships within the input pair proteins. Firstly, DCAPPI generates sequence embedding features using a pre-trained PLM ESM-2 and utilizes predicted 3D structures from the AlphaFold Protein Structure Database to construct residue-level structural graphs. Subsequently, the CCA module fuses the sequence embedding and structural graphs of the input protein pair (*A* and *B*), respectively. Then, the PCA module fetches the interacting context between the protein pair and performs a mutual attention operation for dimension alignment. Finally, based on the success of CollaPPI [11], we use collaborative knowledge from the other 2 protein-related classification tasks, namely, function prediction and location prediction, for enhancing PPI classification performance.

### Protein sequence embedding

Previous PPI prediction studies [12–16,18] mainly utilize sequence-derived features such as evolutionary information, physicochemical properties, intrinsic disorder tendencies, and predicted structure information. Recent approaches increasingly employ pre-trained PLMs, which leverage self-supervised learning on billion-scale protein sequences, aiming to generate semantically rich embeddings. Specifically, pre-trained PLMs such as ESM-2 are capable of capturing residue-level physicochemical properties and pairwise interaction patterns within protein sequences by modeling coevolutionary constraints across diverse proteomes [55]. Among these methods, protein amino acid sequences can be recognized as sentences in natural language processing, where individual amino acids function as semantic units. In our framework, a protein sequence of length *l* is embedded into a feature matrix $S\in R^{l*d}$ which can be shown as follows:

$$S=\left[r_{1},\;r_{2},\;r_{3},\;\cdots ,\;r_{i},\;\cdots ,\;{r_{l}}^{T}\right],\;r_{i}\in R^{d}$$ (1)

where $r_{i}$ represents the *i*th residue in the protein sequence and *d* is the feature embedding dimension of a residue.

### Protein structure representation

Structure-based PPI prediction methods typically model proteins as graphs, where residues serve as nodes and spatial relationships define edges, making this representation suitable for neural network processing. Capitalizing on the AlphaFold database [56], predicted structures for over 200 million proteins across 48 complete proteomes are accessible. Building on this resource, contact maps of any protein can be derived directly from predicted structures. Therefore, those computational approaches can facilitate multiple ways of protein graph construction from atomic spatial coordinates, with each method highlighting different aspects of protein geometry [57]. For proteins without precomputed structures in the AlphaFold database, we use ColabFold [58] as a fallback strategy. ColabFold is an efficient open-source implementation of AF2, capable of generating reliable structural models through automatic construction of multiple sequence alignment.

In this study, we represent a protein structure as a graph $G=\left(V,\;E,\;S\right)$, which models residues as nodes *V*, residue contact relations as edges *E*, and residue embeddings as node features *S*. Specifically, for a protein with a predicted structure, the Euclidean distance between residue pairs is computed based on the spatial coordinates of their $C_{\alpha }$ atoms. Residue pairs with distances $d_{i,j}\leq t$ (where *t* denotes a predefined distance threshold) are classified as interacting, thereby generating a complete binary contact map. The threshold is selected among a set of (4 Å, 6 Å, 8 Å, 10 Å) as a model hyperparameter through 5-fold cross-validation.

### CCA module

Due to the great achievements of protein structure prediction models [41–44], nearly all accessible protein sequences can be assigned corresponding structures. Thus, an increasing number of computational PPI prediction methods exploit hybrid features derived from protein sequences and structures. However, the interaction between proteins is mediated by a complex spatial environment, which is derived from related sequential and spatial neighboring residues. Usually, hybrid methods simply employ direct feature concatenation or treat sequence features as node embedding, which are integrated into GNN-based neural networks. Although these typical techniques can achieve better performance, they ignore the critical information governing the protein interaction through sequential and spatial neighbors interacting.

**Fig. 2.**
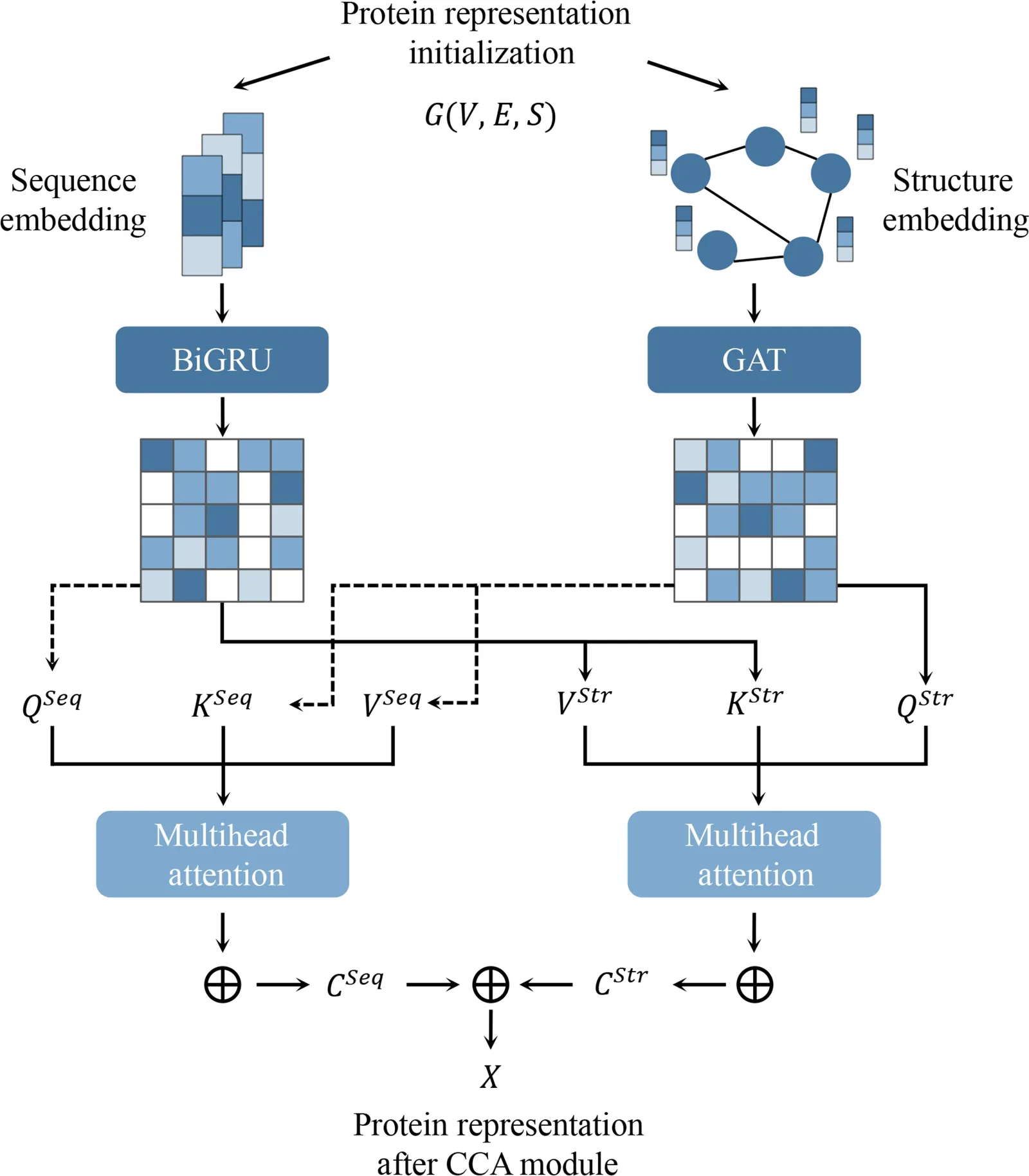
The details of the Channel Cross-Attention (CCA) module. This module separately processes sequence channel features and structure channel features using bidirectional gated recurrent unit (BiGRU) and graph attention network (GAT), respectively. It then employs multihead cross-attention to effectively integrate the sequence and structure embeddings.

As shown in Fig. 1, the dual cross-attention applied to DCAPPI includes a CCA module for sequence–structure feature fusion and a PCA module for protein–partner feature integration. Figure 2 shows the details of the CCA module, which performs standard multihead cross-attention fusion between sequence context representations encoded by BiGRU and spatial topology representations encoded by GAT. Starting from shared ESM residue embeddings, the 2 branches capture linear sequential patterns and 3D spatial adjacency patterns, respectively, providing nonredundant information for PPI prediction. Our contribution lies in their hierarchical organization and biologically motivated application. Moreover, it should be emphasized that although the initial node features of both branches are derived from ESM-2 embeddings, they encode complementary biological information through completely different aggregation methods. The sequence branch captures linear context, while the structural branch extracts spatial topological information. Therefore, the CCA module performs fusion of heterogeneous representations rather than weighting redundant features.

It can be seen from Fig. 2 that the protein feature representation after initialization is expressed as $G=\left(V,\;E,\;S\right)$. In the sequence channel, we utilize the gated recurrent units (GRUs) for feature extraction, which is fed by the protein sequence feature representation *S* embedded by the PLM. For capturing both forward and backward contextual information within protein sequences, we use the BiGRU in the CCA module. The calculations of the GRU are shown as follows:

$$\vec{h_{t}}=\mathrm{GRU}\left(\left[\vec{h_{t-1}},\;S\right]\right)$$ (2)

$$\overset{\leftarrow }{h_{t}}=\mathrm{GRU}\left(\left[\overset{\leftarrow }{h_{t-1}},\;S\right]\right)$$ (3)

where $\vec{h_{t}}$ stands for the forward output and $\overset{\leftarrow }{h_{t}}$ stands for the backward output. The further representation of the sequence channel $C^{\mathrm{Seq}}$ is represented by concatenating $\vec{h_{t}}$ and $\overset{\leftarrow }{h_{t}}$:

$$C^{\mathrm{Seq}}=\left[\vec{h_{t}}\|\overset{\leftarrow }{h_{t}}\right]$$ (4)

In our framework, the protein spatial structure is formally represented as a graph $G=\left(V,\;E,\;S\right)$, where *V* denotes the set of nodes (corresponding to amino acid residues with length *l*) and $E\in R^{l*l}$ defines the adjacency matrix derived from a predicted contact map. The matrix *E* encodes pairwise spatial connections between residues, serving as the topological foundation for structural feature extraction. In order to capture hierarchical structural dependencies in the protein structure channel, we employ a one-layer GAT with *K* attention heads in the CCA module. GAT leverages attention mechanisms to dynamically weight neighbor node contributions during feature aggregation, prioritizing spatially influential residues even when sequentially distant. The initial node features $S={\left[r_{1},\;r_{2},\;r_{3},\;\cdots ,\;r_{i},\;\cdots ,\;r_{l}\right]}^{T},\;r_{i}\in R^{d}$, are generated from the PLM, which is the same as the input in the sequence channel. The *i*th node $r_{i}$ aggregates features from its first-order neighbors $N_{i}$, and the updated feature matrix $r_{i}^{\prime }$ of the *i*th node is calculated as follows:

$$r_{i}^{\prime }=\sigma \left(\frac{1}{K}\sum_{k=1}^{K}\sum_{j\in N_{i}}\alpha_{\mathrm{ij}}^{k}W^{k}r_{j}\right)$$ (5)

where σ is the ReLU activation function, $\alpha_{\mathrm{ij}}^{k}$ means the normalized attention coefficient for neighbor $r_{j}$ computed by the *k*th attention head, and $W^{k}$ denotes the learnable weight matric for the *k*th attention head. The formula for calculating $\alpha_{\mathrm{ij}}^{k}$ is shown as follows:

$$\alpha_{\mathrm{ij}}^{k}=\frac{\exp\left(\text{LeakyReLU}\left(a^{k^{T}}\left[W^{k}r_{i}\|W^{k}r_{j}\right]\right)\right)}{\sum_{m\in N_{i}}\exp\left(\text{LeakyReLU}\left(a^{k^{T}}\left[W^{k}r_{i}\|W^{k}r_{m}\right]\right)\right)}$$ (6)

where ‖ denotes matrix concatenation, $a^{k^{T}}$ is a learnable weight matrix of the *k*th attention head, *T* means the matrix transposition, and *W* is a learnable parameter for the accurate calculation of the attention coefficient. The further representation of the structure channel $C^{\mathrm{Str}}$ is represented as the concatenation of all residues shown as follows:

$$C^{\mathrm{Str}}={\left[{r_{1}}^{\prime },\;{r_{2}}^{\prime },\;{r_{3}}^{\prime },\;\cdots ,\;{r_{i}}^{\prime },\;\cdots ,\;{r_{l}}^{\prime }\right]}^{T}$$ (7)

For feature fusion between the sequence and structure channels, we built multihead cross-attention networks with an attention head number of *h*. The multihead cross-attention network employs *h* parallel attention heads to model inter-protein interactions between the protein sequence and structure channels embedding $C^{\mathrm{Str}}$ and $C^{\mathrm{Seq}}$. For the sequence channel embedding $C^{\mathrm{Seq}}$, the query matrix of the *i*th attention head $Q_{i}^{\mathrm{Seq}}$ are computed through linear projections by the following formula:

$$Q_{i}^{\mathrm{Seq}}=C^{\mathrm{Seq}}W_{i}^{Q}\;\left(i=1,\;2,\;\ldots ,\;h\right)$$ (8)

Furthermore, the structure channel embedding $C^{\mathrm{Str}}$ generate key-value pairs via formulas shown as follows:

$$K_{i}^{\mathrm{Seq}}=C^{\mathrm{Str}}W_{i}^{K},\;V_{i}^{\mathrm{Seq}}=C^{\mathrm{Str}}W_{i}^{V}$$ (9)

The attention weight of the *i*th head derived through scaled dot-product operations is shown as

$${\text{AttentionScore}}_{i}^{\mathrm{Seq}}=\text{Softmax}\left(\frac{Q_{i}^{\mathrm{Seq}}{K_{i}^{\mathrm{Seq}}}^{T}}{\sqrt{d}}\right)$$ (10)

where *d* signifies the column dimension of $W_{i}^{Q}$ and *T* means the matrix transposition. The attention weight is multiplied by $V_{i}^{\mathrm{Seq}}$ to produce attention heads that is given by

$${\text{Head}}_{i}^{\mathrm{Seq}}={\text{AttentionScore}}_{i}^{\mathrm{Seq}}V_{i}^{\mathrm{Seq}}$$ (11)

The further embedding of the protein sequence channel is represented by the multihead output, which integrates all heads through concatenation and linear transformation with $W_{O}^{\mathrm{Seq}}$ and is expressed by

$${\left.C^{{\mathrm{Seq}}^{\prime }}=\right\|}_{i=1}^{h}\left({\text{Head}}_{i}^{\mathrm{Seq}},\;\cdots ,\;{\text{Head}}_{h}^{\mathrm{Seq}}\right)W_{O}^{\mathrm{Seq}}$$ (12)

Similarly, the corresponding further embedding of the protein structure channel can be shown as follows:

$${\left.C^{{\mathrm{Str}}^{\;\prime }}=\right\|}_{i=1}^{h}\left({\text{Head}}_{i}^{\mathrm{Str}},\;\cdots ,\;{\text{Head}}_{h}^{\mathrm{Str}}\right)W_{O}^{\mathrm{Str}}$$ (13)

Finally, the protein representation integrating sequence and structure channels through the CCA module is contracted by concatenating $C^{{\mathrm{Seq}}^{\prime }}$ and $C^{{\mathrm{Str}}^{\prime }}$ given by

$$X=C^{{\mathrm{Seq}}^{\;\prime }}\|C^{{\mathrm{Str}}^{\;\prime }}$$ (14)

### PCA module

The computational PPI prediction method takes a pair of proteins as input and judges whether they interact or not. However, the current approaches usually regard the protein pair as separate entities and construct fused features relying on basic operations such as matrix concatenation or element-wise multiplication. This way of feature fusion is oversimple and cannot fully capture the critical information governing the protein–partner interaction relationships.

**Fig. 3.**
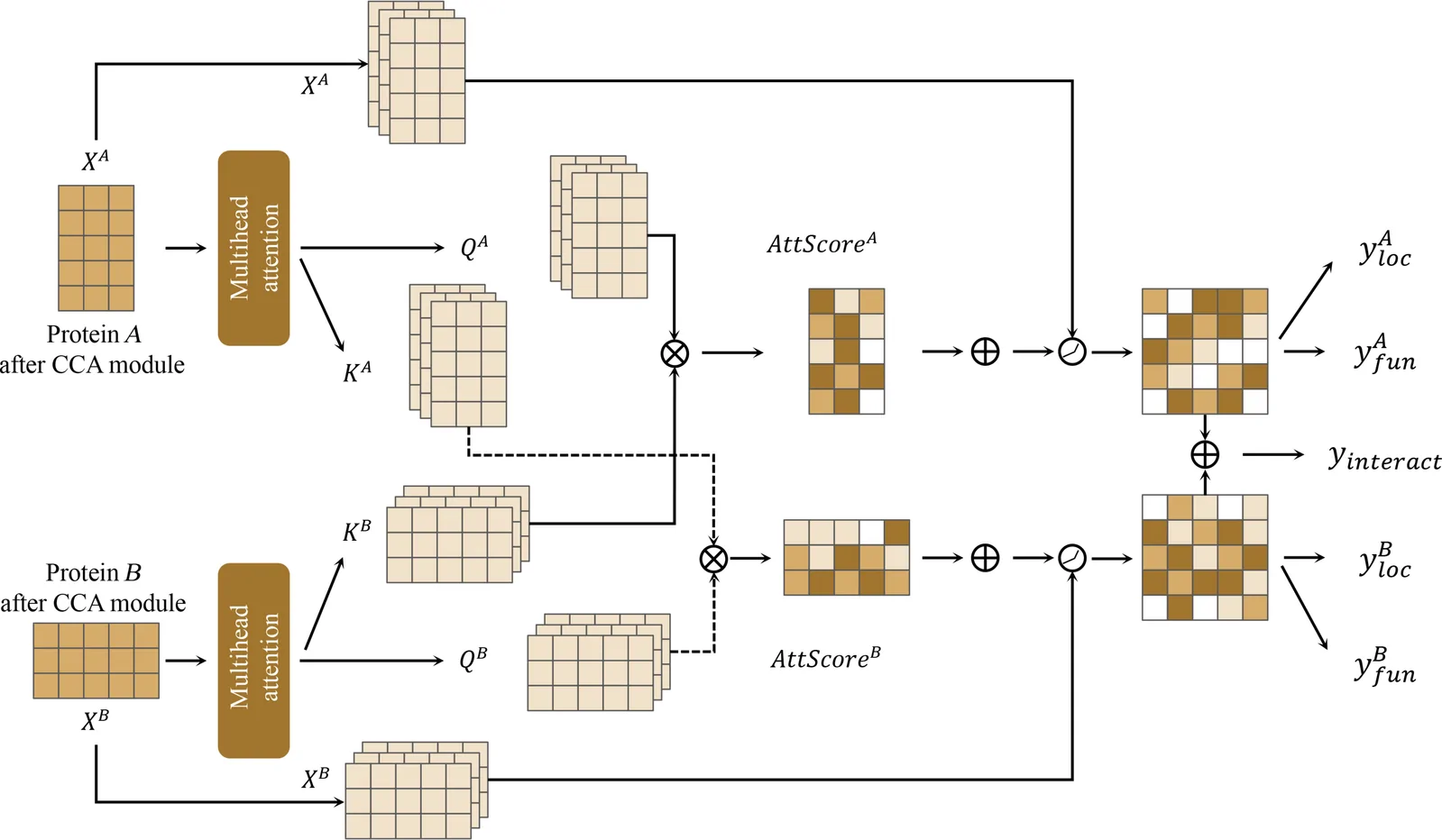
The details of the Partner Cross-Attention (PCA) module. The PCA module takes the input protein *A* and its interacting partner *B* as input, which are represented as $X^{A}$ and $X^{B}$, respectively, after being processed by the Channel Cross-Attention (CCA) module. To implement effective protein–partner feature fusion, we construct multihead cross-attention networks with multiple attention heads.

As shown in Fig. 3, DCAPPI utilizes the PCA module for protein–partner feature fusion at the inter-protein level. The PCA module follows the standard multihead cross-attention mechanism. Its novelty resides in applying it explicitly between the 2 protein feature sets after CCA fusion, rather than on merged features. Figure 3 shows the details of the PCA module, where the input protein *A* and interacting partner *B* after the CCA module are represented as $X^{A}$ and $X^{B}$, shown as follows:

$$X^{A}={\left[x_{1},\;x_{2},\;x_{3},\;\cdots ,\;x_{l},\;\cdots ,\;x_{L_{1}}\right]}^{T}\in L_{1}*R^{d_{\mathrm{CCA}}}$$ (15)

$$X^{B}={\left[x_{1},\;x_{2},\;x_{3},\;\cdots ,\;x_{l},\;\cdots ,\;x_{L_{2}}\right]}^{T}\in L_{2}*R^{d_{\mathrm{CCA}}}$$ (16)

where $L_{1}$ and $L_{2}$ are the protein lengths of proteins *A* and *B*, respectively. $d_{\mathrm{CCA}}$ is the feature dimension generated by the CCA module.

For protein–partner feature fusion, we built multihead cross-attention networks with an attention head number of *m*; the calculation of the attention score for proteins *A* and *B* is given by

$${\left.{\text{AttScore}}^{A}=\right\|}_{n=1}^{m}\text{Softmax}\left(\frac{X^{A}W_{n}^{Q}{\left(X^{B}W_{n}^{K}\right)}^{T}}{\sqrt{d}}\right)$$ (17)

$${\left.{\text{AttScore}}^{B}=\right\|}_{n=1}^{m}\text{Softmax}\left(\frac{X^{B}W_{n}^{Q}{\left(X^{A}W_{n}^{K}\right)}^{T}}{\sqrt{d}}\right)$$ (18)

where *d* is the column dimension of $W_{n}^{Q}$ or $W_{n}^{K}$, *T* denotes the transposition operation, $W_{n}^{Q}$ and $W_{n}^{K}$ are learnable parameters, and ‖ means matrix concatenation.

It should be noted that ${\text{AttScore}}^{A}$ and ${\text{AttScore}}^{B}$ are with the shape of $L_{1}*L_{2}$ and $L_{2}*L_{1}$, where $L_{1}$ and $L_{2}$ are the lengths of the proteins *A* and *B*, respectively. They can be expressed as

$${\text{AttScore}}^{A}=\left\{p_{11},\;p_{12},\;\cdots ,\;p_{L_{1}*L_{2}}\right\}$$ (19)

$${\text{AttScore}}^{B}=\left\{q_{11},\;q_{12},\;\cdots ,\;q_{L_{2}*L_{1}}\right\}$$ (20)

The interacting knowledge mechanism processes the attention weight matrix from the perspective of the first protein (with $L_{1}$ residues) by computing column-wise averages, generating an intermediate residue important vector $p_{i}^{\prime }$. This vector undergoes SoftMax normalization to yield a probabilistic residue weighting $a_{i}$, where each element quantifies the relative functional importance of its corresponding residue. The context-aware embedding $x_{i}^{\prime }$ is then constructed as the weighted sum of the protein’s residue-level embeddings, using $a_{i}$ as the weighting coefficients. An identical procedure is applied to protein *B* to derive $x_{j}^{\prime }$. The interacting knowledge mechanism procedures are calculated as follows:

$$p_{i}^{\prime }=\frac{1}{L_{2}}\sum_{j=1}^{L_{2}}p_{\mathrm{ij}},\;q_{j}^{\prime }=\frac{1}{L_{1}}\sum_{i=1}^{L_{1}}q_{\mathrm{ji}}$$ (21)

$$a_{i}=\frac{\exp\left(p_{i}^{\prime }\right)}{\sum_{k=1}^{L_{1}}\exp\left(p_{k}\right)},\;a_{j}=\frac{\exp\left(q_{j}^{\prime }\right)}{\sum_{k=1}^{L_{2}}\exp\left(q_{k}\right)}$$ (22)

$${x_{i}}^{\prime }=\sum_{i=1}^{L_{1}}a_{i}x_{i},\;{x_{j}}^{\prime }=\sum_{j=1}^{L_{2}}a_{j}x_{j}$$ (23)

By calculating the column average of cross-protein attention weights, we obtain the overall importance score of each residue to its interacting partner, thereby achieving context-aware feature aggregation.

### Multitask collaboration

Conventional PPI prediction frameworks directly feed protein pair representations $X_{A}$ and $X_{B}$ into fully connected classifiers for binary interaction classification. In order to improve the prediction performance of the model, we introduce a homogeneous MTC module with 2 auxiliary tasks, namely, protein function prediction and subcellular localization prediction. This operation is inspired by CollaPPI [11] and motivated by the established biological principles of functional coherence and spatial proximity. Moreover, this module is built upon the standard hard parameter-sharing paradigm, which is a widely adopted framework in bioinformatics studies. Functional coherence refers to the phenomenon that proteins sharing identical functions display a higher interaction propensity, since they are jointly involved in common biological processes [59]. Spatial proximity describes that proteins located within the same subcellular compartments exhibit a higher interaction likelihood, as their colocalization facilitates potential binding [60,61].

In the function prediction task, Gene Ontology (GO) terms annotating protein functions are curated from UniProt. The 100 most frequent GO terms form a fixed vocabulary, mitigating dimensionality explosion. Proteins are mapped to this vocabulary via a binary bag-of-words representation, generating a 100-dimensional label vector $y_{\mathrm{go}}\in {\left\{0,\;1\right\}}^{100}$, where 1 indicates function presence. In the subcellular localization task, subcellular annotations undergo identical vocabulary-based encoding, producing a 100-dimensional label $y_{\mathrm{loc}}\in {\left\{0,\;1\right\}}^{100}$. Both tasks are treated as multilabel classification problems to accommodate proteins with multiple functions/locations. The MTC module employs a hard parameter-sharing backbone with 3 task-specific tower classifiers, which are shown as follows:

$$y_{\mathrm{go}}^{A}=W_{G}^{A}{x_{i}}^{\prime }+b_{G}^{A},\;y_{\mathrm{go}}^{B}=W_{G}^{B}{x_{j}}^{\prime }+b_{G}^{B}$$ (24)

$$y_{\mathrm{loc}}^{A}=W_{L}^{A}{x_{i}}^{\prime }+b_{L}^{A},\;y_{\mathrm{loc}}^{B}=W_{L}^{B}{x_{j}}^{\prime }+b_{L}^{B}$$ (25)

$$y_{\text{interact}}=W_{I}\left[{x_{i}}^{\prime },\;{x_{j}}^{\prime }\right]+b_{I}$$ (26)

It is worth noting that although Eqs. (24) and (25) define independent classifier weights ($W_{G}^{A},W_{G}^{B}$) for proteins *A* and *B*, respectively, this applies only to different auxiliary tasks. For the same auxiliary task, proteins *A* and *B* share exactly the same classifier weights, ensuring the parameter symmetry of the Siamese network and the order independence of prediction.

Each tower consists of stacked fully connected layers. This design enables cross-task knowledge transfer that functional and localization cues guide and refine interaction prediction, while interaction signals in turn enhance the representation of function and subcellular localization. The model generates predictive outputs for 3 distinct tasks: protein function classification, subcellular localization, and binary PPI prediction. Each of task-specific losses $L_{\mathrm{go}}$, $L_{\mathrm{loc}}$, and $L_{\text{interact}}$ is computed via binary cross-entropy. To dynamically balance loss scales across tasks during training, we implement an improved uncertainty-weighted loss mechanism, departing from static summation or predefined weighting schemes. The total loss is defined as

L=12T12Lgo+12T22Lloc+12T32Linteract+log1+T12+log1+T22+log1+T32 (27)

## Experiment Setup

### Datasets

In this study, we utilize 3 benchmark datasets, namely, the Yeast, Multi-species, and Multi-class datasets, to evaluate the performance of our proposed method. Comprehensive statistical descriptions of these datasets are provided in Table 1 and described as follows.

**Table 1.** Summary of the datasets

Dataset	Protein	Positive samples	Negative samples
Yeast	2,497	5,594	5,594
Multi-species (All)	11,529	32,959	32,959
Multi-species (<40%)	9,739	25,916	22,012
Multi-species (<25%)	7,790	19,458	15,827
Multi-species (<10%)	5,769	12,641	9,819
Multi-species (<1%)	5,171	10,747	8,065
Multi-class	4,577	75,875	
Gold standard dataset (train)	4,286	81,596	81,596
Gold standard dataset (val)	3,711	29,630	29,630
Gold standard dataset (test)	3,022	26,024	26,024

The Yeast dataset comprises 5,594 interacting (positive) and 5,594 noninteracting (negative) protein pairs derived from 2,497 proteins. Positive samples in this dataset comprise exclusively experimentally verified physical PPIs, ensuring high-confidence annotations. Conversely, negative samples are generated by random pairing of proteins without documented interaction evidence, adhering to standard practice for noninteracting pair construction in PPI benchmarks. Proteins with fewer than 50 amino acids or ≥40% sequence identity are excluded. We use 5-fold cross-validation on this dataset, and the segmentation strategy is based on random pair-level splitting of protein pairs. Although this is a common practice in many works in this field, we acknowledge that it may lead to protein identity leakage. Therefore, we also adopt stricter experiments on the gold standard datasets [62] to verify the true generalization ability of the model.

The Multi-species dataset integrates proteins from *Escherichia coli*, *Caenorhabditis elegans*, and *Drosophila melanogaster* to assess PPI prediction performance. All proteins exceed 50 amino acids in length. To evaluate model robustness under low-sequence-identity conditions, the dataset is partitioned into subsets based on sequence identity thresholds of 1%, 10%, 25%, and 40%, with the whole dataset (all) retained for benchmarking comparative analysis.

The Multi-class dataset comprises 4,577 human proteins annotated with 7 interaction types: binding, catalysis, reaction, posttranslational modification, inhibition, expression, and activation. This dataset is used to further evaluate the method’s capability beyond binary interaction prediction.

To rigorously evaluate the true generalization ability of DCAPPI and rule out performance inflation caused by dataset biases such as data leakage, sequence homology shortcuts and node-degree artifacts, we conduct additional validation on the gold standard PPI dataset proposed by Bernett *et al*. [62]. This dataset is built from the HIPPIE v2.3 database. The human proteome is partitioned into 3 disjoint subsets via the KaHIP algorithm with minimized cross-subset sequence similarity, ensuring zero protein overlap among the training, validation, and test sets. Negative samples are generated by a degree-preserving sampling strategy to eliminate node-degree shortcuts, and sequences within each subset are further redundancy-reduced by CD-HIT at a 40% identity threshold.

### Evaluation metrics

PPI prediction constitutes a binary classification problem, where interacting pairs are classified as positive instances and noninteracting pairs as negative instances. To comprehensively evaluate the performance of DCAPPI, 6 metrics are employed, namely, accuracy (ACC), precision (PRE), sensitivity (SEN), specificity (SPE), F1-score (F1), and Matthews correlation coefficient (MCC), which are defined as follows:

$$\mathrm{ACC}=\frac{\mathrm{TP}+\mathrm{TN}}{\mathrm{TP}+\mathrm{FN}+\mathrm{TN}+\mathrm{FP}}$$ (28)

$$\mathrm{PRE}=\frac{\mathrm{TP}}{\mathrm{TP}+\mathrm{FP}}$$ (29)

$$\mathrm{SEN}=\frac{\mathrm{TP}}{\mathrm{TP}+\mathrm{FN}}$$ (30)

$$\mathrm{SPE}=\frac{\mathrm{TN}}{\mathrm{TN}+\mathrm{FP}}$$ (31)

$$F1=\frac{2\times \mathrm{PRE}\times \mathrm{SEN}}{\mathrm{PRE}+\mathrm{SEN}}$$ (32)

$$\mathrm{MCC}=\frac{\mathrm{TP}\times \mathrm{TN}-\mathrm{FP}\times \mathrm{FN}}{\sqrt{\left(\mathrm{TP}+\mathrm{FP}\right)\left(\mathrm{TP}+\mathrm{FN}\right)\left(\mathrm{TN}+\mathrm{FP}\right)\left(\mathrm{TN}+\mathrm{FN}\right)}}$$ (33)

where TP (true positive) is the number of interacting protein pairs that are correctly predicted as interacting, FP (false positive) is the number of noninteracting protein pairs that are falsely predicted as interacting, TN (true negative) denotes the number of noninteracting protein pairs that are identified correctly, and FN (false negative) denotes the number of interacting protein pairs that are identified falsely. The metrics above are all threshold dependent, which depends on the threshold for judging the positive and negative predictions. We set this threshold to 0.5 as the comparative method.

### Settings of hyperparameters

DCAPPI is implemented using PyTorch, adopting a weighted loss function and the Adaptive Moment Estimation (Adam) optimizer for training. After hyperparameter tuning, the final loss weights for the main task (PPI prediction), functional prediction, and subcellular localization prediction were determined to be 5.84, 1.27, and 2.85, respectively. Learning rates of 0.1, 0.01, 0.001, and 0.0001 are tested, along with training batch sizes of 16, 32, and 64 and dropout rates of 0.2, 0.5, and 0.8. Through experimentation, the final hyperparameters are determined as a learning rate of 0.001, a training batch size of 32, and a dropout rate of 0.5. To evaluate the impact of the number of attention heads, different numbers of heads (1, 2, and 4) are tested in the CCA and PCA modules when processing input proteins *A* and *B*, resulting in various combinations. For testing the representational capabilities of different language models, 3 PLMs are evaluated: ProtBert, ProtT5, and ESM-2. Meanwhile, to assess the quality of protein contact maps, different distance thresholds (4, 6, 8, and 10 Å) are employed. DCAPPI is trained on a single NVIDIA RTX 4090 graphics processing unit with a maximum of 200 epochs. The training time per epoch ranges from approximately 3 to 25 min, depending on the dataset size.

## Results

### Impact of PLMs

PLMs have emerged as powerful tools for extracting meaningful biological information from massive protein sequence databases. By leveraging self-supervised learning on billions of protein sequences, PLMs capture intricate patterns related to protein structure, function, and evolutionary relationships. The high-dimensional embeddings generated by PLMs serve as foundational inputs for a wide range of downstream tasks, including protein structure prediction, functional annotation, and PPI prediction. However, variations in PLMs architectures and training objectives lead to marked differences in the quality and expressiveness of the resulting protein embeddings. These differences directly influence the performance of downstream PPI prediction models, as the embeddings form the basis for subsequent feature extraction and fusion.

**Fig. 4.**
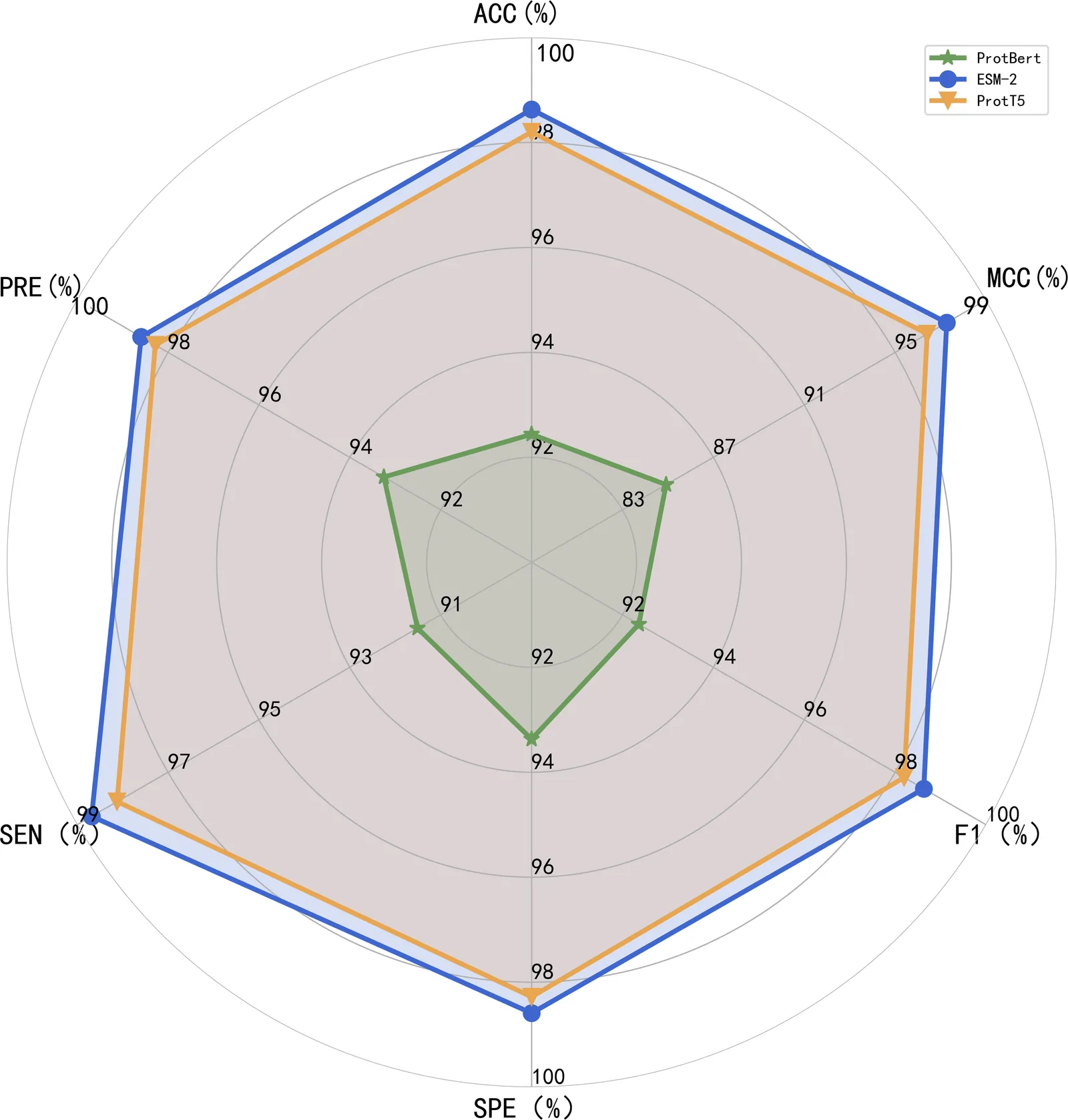
The impact of different protein language models (PLMs).

To systematically evaluate the impact of PLM selection on DCAPPI’s performance, we conduct comparative experiments on the Yeast dataset using 3 state-of-the-art PLMs: ProtBert, ProtT5, and ESM-2. The experimental results are presented in Fig. 4.

As illustrated in Fig. 4, DCAPPI achieves the best performance across all evaluation metrics when using ESM-2 as the protein sequence embedding generator. ESM-2 outperforms ProtBert by 6.2% in ACC, 6.27% in F1, and 12.36% in MCC, demonstrating its superior ability to capture residue-level semantic and structural features. Compared to ProtT5, ESM-2 improves ACC by 4.3%, F1 by 4.3%, and MCC by 0.86%, highlighting its advantage in modeling coevolutionary constraints and long-range dependencies within protein sequences.

The superior performance of ESM-2 can be attributed to its unique design and training paradigm. ESM-2 is trained on a massive dataset of 250 million protein sequences, enabling it to learn deep evolutionary patterns and context-aware residue interactions that are critical for PPI prediction. Unlike traditional PLMs that focus primarily on sequence-level patterns, ESM-2 effectively models the coevolutionary signals between amino acids, which directly reflect structural and functional constraints governing protein interactions. Additionally, ESM-2’s transformer-based architecture with optimized depth and attention mechanisms enhances its capacity to capture both local sequence motifs and global structural features, providing a more comprehensive representation of protein sequences for subsequent fusion via DCAPPI’s cross-attention modules. In contrast, ProtBert and ProtT5, while powerful in their own right, exhibit relatively limited performance in this study.

Based on these results, ESM-2 is selected as the default PLM for DCAPPI. Its ability to generate high-quality, biologically meaningful embeddings lays a solid foundation for the subsequent intra-protein (CCA module) and inter-protein (PCA module) feature fusion, ultimately contributing to DCAPPI’s superior predictive performance. This finding underscores the importance of selecting a PLM that is specifically optimized for capturing the evolutionary and structural cues relevant to the target downstream task and provides valuable insights for future PPI prediction model development.

### Impact of distance thresholds

Protein contact maps serve as the structural foundation for modeling protein 3D interactions in DCAPPI, where residues are represented as nodes and spatial proximity between residues is encoded as edges. Specifically, an edge is defined between 2 residues if the Euclidean distance between their *α*-carbon atoms is less than a predefined threshold. The choice of this distance threshold directly shapes the topological structure of the contact map. Overly strict thresholds may exclude functionally critical interaction residues, while excessively loose thresholds can introduce irrelevant edges that obscure true structural patterns. Both scenarios degrade the quality of structural feature extraction and subsequent inter-protein interaction modeling.

**Fig. 5.**
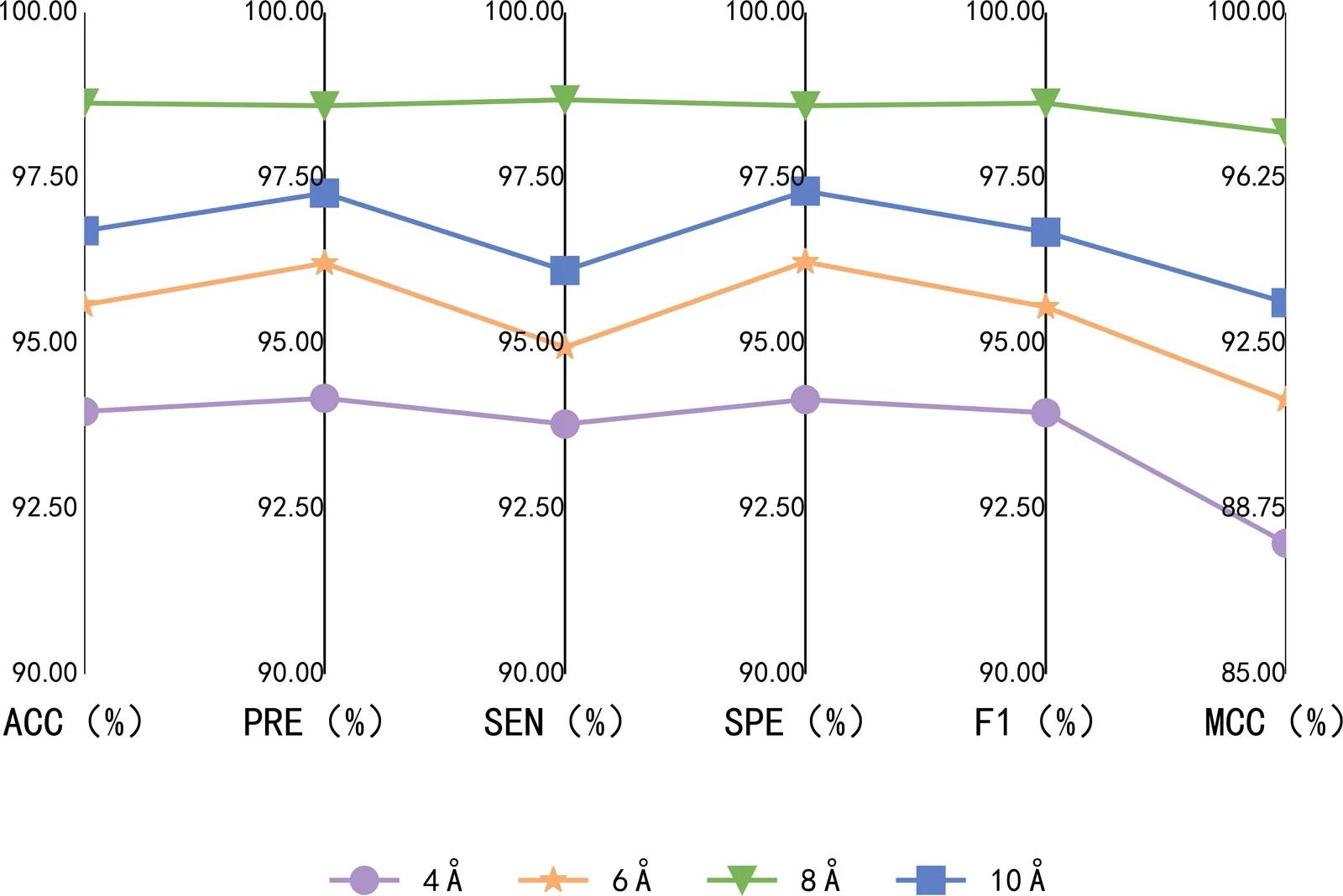
The impact of distance thresholds on protein contact map construction.

To identify the optimal distance threshold for contact map construction, we conduct experiments on the Yeast dataset using 4 biologically relevant threshold values: 4, 6, 8, and 10 Å. These thresholds are selected based on prior literature [11] and biological insights. Residues within 8 to 10 Å are generally considered to be in spatial proximity, while distances below 4 Å typically indicate direct atomic interactions. A 5-fold cross-validation strategy is employed to ensure the robustness of the results, and the experimental results are presented in Fig. 5.

As shown in Fig. 5, DCAPPI achieves the best overall performance across all evaluation metrics when the distance threshold is set to 8 Å. At 8 Å, the model attains an ACC of 98.63% and an MCC of 97.27%, outperforming the 4-Å threshold by 4.66% in ACC and 9.30% in MCC. Compared to the 6-Å threshold, the 8-Å setting improves ACC by 3.05% and MCC by 6.05%, indicating that a moderately larger threshold captures more contextually relevant structural interactions. When the threshold is increased to 10 Å, performance declines; e.g., ACC drops by 1.93% and MCC decreases by 3.85% compared to those of 8 Å. This indicates that the inclusion of distant, noninteracting residues introduces noise into the structural graph, diluting the signal of true interaction sites.

The superior performance of the 8-Å threshold can be attributed to its balance between specificity and coverage in capturing biologically meaningful structural interactions. Residues within 8 Å of each other are likely to be part of the same structural domain or functional interface, providing critical spatial cues for PPI prediction. This threshold effectively includes residues involved in indirect interactions that are essential for stabilizing protein complexes. The 8-Å threshold avoids the overconstraint of smaller thresholds, which often miss spatially adjacent residues that contribute to the overall structural context of interaction sites. For example, residues in the vicinity of an active site may play a role in shaping the binding pocket, and their inclusion enriches the structural feature representation. Unlike the 10-Å threshold, 8 Å minimizes the inclusion of irrelevant residues from distant structural regions, reducing noise in the contact map and ensuring that the GAT layer in the CCA module focuses on functionally relevant spatial dependencies.

These results align with prior studies [11] that highlight 8 Å as an optimal threshold for balancing structural relevance and noise reduction in contact map construction. The 10-Å threshold, while capturing a broader range of spatial relationships, introduces redundant edges that increase computational complexity and hinder the model’s ability to prioritize key interaction residues. In contrast, smaller thresholds fail to capture the full scope of structural context required for accurate PPI prediction, as protein interactions often involve larger structural interfaces rather than isolated atomic contacts.

Based on the findings mentioned above, the distance threshold on contact map construction in DCAPPI is fixed at 8 Å. This configuration ensures that the structural graph accurately reflects biologically meaningful spatial relationships, providing a robust foundation for the CCA module to integrate sequence and structural features and ultimately enhancing the model’s ability to predict PPIs. This result underscores the importance of tailoring structural preprocessing steps to the biological constraints of the target task, as improper parameterization can impact downstream model performance.

### Impact of the number of attention heads

Multihead attention is a pivotal component in DCAPPI, employed across multiple modules to capture diverse feature representations. The number of attention heads directly influences the model’s capacity to learn complex patterns, computational efficiency, and training stability. Fewer heads may limit the model’s ability to capture varied feature interactions, while an excessive number can lead to overfitting and increased computational overhead. This section evaluates the optimal configuration of attention heads through systematic experiments.

To systematically assess the effect of attention head counts, we first encode the positions of multihead attention layers in DCAPPI using a 4-digit code. Each digit in the code corresponds to the number of heads at a specific module. Digit 1 means the number of heads in the GAT layer for protein structure feature extraction. Digits 2 and 3 denote the number of heads in the cross-attention layer for fusing sequence and structure features, respectively. Digit 4 stands for the number of heads in the PCA module for fusing target protein and partner protein features. We test various combinations of heads (1, 2, 4) across these positions to identify the optimal configuration.

Table 2 presents the performance of DCAPPI with different attention head configurations on the Yeast dataset. The results indicate that the configuration encoded as 2224 achieves the best overall performance, with the highest values in all metrics. Using 2 heads for the structure feature extraction balances the granularity of structural feature capture without high computational cost. Employing 2 heads for sequence and structure cross-attention layers effectively integrates complementary information from different modalities. Using 4 heads for partner protein fusion in the PCA module enhances the model’s ability to capture complex inter-protein interaction patterns, which is critical for distinguishing true PPIs from false positives.

**Table 2.** Results of different numbers of attention heads in multihead attention mechanisms. Note: The best results are shown in bold.

Number of heads	ACC (%)	PRE (%)	SEN (%)	SPE (%)	F1 (%)	MCC (%)
1111	98.45 ± 0.34	**98.64 ± 0.36**	98.27 ± 0.37	**98.64 ± 0.36**	98.45 ± 0.34	96.91 ± 0.69
1112	98.49 ± 0.19	98.59 ± 0.25	98.39 ± 0.26	98.59 ± 0.25	98.49 ± 0.19	96.98 ± 0.37
1222	98.55 ± 0.21	98.56 ± 0.52	98.55 ± 0.39	98.56 ± 0.54	98.55 ± 0.21	97.11 ± 0.42
2222	98.52 ± 0.11	98.60 ± 0.31	98.44 ± 0.32	98.61 ± 0.32	98.52 ± 0.11	97.05 ± 0.22
2224 (DCAPPI)	**98.63 ± 0.20**	98.59 ± 0.36	**98.68 ± 0.31**	98.59 ± 0.037	**98.63 ± 0.20**	**97.27 ± 0.40**
2444	94.19 ± 0.67	93.75 ± 1.59	94.74 ± 1.36	93.64 ± 1.77	94.22 ± 0.65	88.42 ± 1.34
4444	94.79 ± 0.58	94.92 ± 0.47	94.65 ± 1.46	94.92 ± 0.55	94.78 ± 0.62	89.59 ± 1.16

ACC, accuracy; PRE, precision; SEN, sensitivity; SPE, specificity; F1, F1-score; MCC, Matthews correlation coefficient; DCAPPI, Dual Cross-Attention network for Protein-Protein Interaction prediction

The superior performance of the 2224 configuration highlights the importance of tailoring attention head counts to specific tasks. A lower number of attention heads may insufficiently capture diverse feature interactions, leading to suboptimal representation learning. Moderate attention heads strike a balance between feature diversity and computational efficiency, making them suitable for feature extraction and fusion. Higher head counts in the PCA module enable more fine-grained modeling of inter-protein dependencies, leveraging the increased capacity to capture coevolutionary signals and binding motifs. This sensitivity to excessive attention heads stems from the inherent sparsity of PPI signals. Only a few residues mediate true binding, and too many heads can scatter limited signals into too many subspaces, introducing noise from noninterface regions and diluting attention to truly binding residues. These findings provide insights into the design of multihead attention mechanisms for PPI prediction, emphasizing the need for task-specific optimization of hyperparameters to maximize model performance.

### Ablation studies

To thoroughly validate the performance of DCAPPI and systematically evaluate the contribution of intra-protein and inter-protein feature extraction, as well as the effectiveness of multitask learning, this section dissects the core modules of our proposed model. DCAPPI incorporates 2 specialized cross-attention mechanisms. One is the CCA module, which is designed to capture intra-protein sequence–structure dependencies. The other is the PCA module, which is responsible for fusing features between the target protein and its interaction partner. Moreover, the MTC module is built for leveraging cross-task knowledge transfer. Ablation experiments on the Yeast dataset were designed to isolate and quantify the individual contributions of these modules.

Five ablation variants are constructed by removing key components while keeping the rest of the architecture identical to the full model. The No-SEQ variant removes the sequence encoding pathway and retains only the GAT structural representation branch built on contact maps. The No-STR variant removes the structure encoding pathway and retains only the BiGRU-based sequence representation branch. The No-CCA variant removes the CCA module. After extracting sequence features via BiGRU and structure features via GATs, these feature matrices are directly concatenated without cross-attention interaction. This variant tests the necessity of CCA for integrating complementary intra-protein information. The No-PCA variant removes the PCA module. Following intra-protein feature extraction, the target protein’s features and its partner’s features are directly concatenated without cross-attention fusion. This variant evaluates the impact of the PCA module on inter-protein feature aggregation. Finally, the No-MTC variant ablates the MTC knowledge. After completing feature fusion within and between proteins, it makes direct probability predictions based on the output embeddings, without leveraging auxiliary task knowledge. This variant assesses the value of biological knowledge assistance from the MTC module on prediction performance.

Experimental results are shown in Table 3, which demonstrate that all ablation variants exhibit marked performance degradation across all metrics compared to the full DCAPPI model. Specifically, the No-SEQ variant (structure-only) suffers the most severe performance degradation, with ACC dropping to 93.89% and MCC to 87.78%. The No-STR variant (sequence-only) achieves an ACC of 97.63% and an MCC of 95.27%. Despite outperforming the structure-only variant, it still falls behind the full model by 1.00 percentage point in ACC and 2.00 percentage points in MCC. The No-CCA variant shows reduced ACC by 3.11% and MCC by 6.21%, indicating that CCA effectively integrates complementary sequence and structure information to enhance intra-protein feature representation. The No-PCA variant performs the worst, with a 4.21% drop in ACC and an 8.57% decrease in MCC. This highlights the critical role of PCA in capturing inter-protein interaction patterns, as direct feature concatenation fails to model complex dependencies between binding partners. The No-MTC variant shows the least severe performance decline, with ACC reduced by 0.87% and MCC by 1.75%.

**Table 3.** The performance of DCAPPI and its variants. Note: The best results are shown in bold.

Ablation variants	ACC (%)	PRE (%)	SEN (%)	SPE (%)	F1 (%)	MCC (%)
No-SEQ	93.89 ± 0.01	94.27 ± 0.01	93.46 ± 0.01	94.32 ± 0.01	93.86 ± 0.01	87.78 ± 0.01
No-STR	97.63 ± 0.00	98.04 ± 0.01	97.21 ± 0.00	98.05 ± 0.01	97.62 ± 0.00	95.27 ± 0.01
No-CCA	95.52 ± 0.01	95.89 ± 0.01	95.14 ± 0.01	95.91 ± 0.01	95.51 ± 0.01	91.06 ± 0.01
No-PCA	94.32 ± 0.01	94.04 ± 0.01	94.69 ± 0.02	93.96 ± 0.02	94.34 ± 0.01	88.70 ± 0.02
No-MTC	97.76 ± 0.00	97.73 ± 0.01	97.78 ± 0.01	97.73 ± 0.01	97.76 ± 0.00	95.52 ± 0.01
DCAPPI	**98.63 ± 0.20**	**98.59 ± 0.36**	**98.68 ± 0.31**	**98.59 ± 0.04**	**98.63 ± 0.20**	**97.27 ± 0.40**

DCAPPI, Dual Cross-Attention network for Protein-Protein Interaction prediction; ACC, accuracy; PRE, precision; SEN, sensitivity; SPE, specificity; F1, F1-score; MCC, Matthews correlation coefficient; No-SEQ, structure-only; No-STR, sequence-only; No-CCA, removes the Channel Cross-Attention module; No-PCA, removes the Partner Cross-Attention module; No-MTC, ablates the multitask collaboration knowledge

The superior performance of the full model underscores 3 key insights. Firstly, for intra-protein feature integration, the CCA module’s cross-attention mechanism enables the model to prioritize functionally relevant residues by leveraging both sequential context and structural proximity. Secondly, for inter-protein feature aggregation, the PCA module facilitates the discovery of coevolutionary signals and binding motifs between interacting proteins, which is essential for distinguishing specific PPIs from nonspecific interactions. Finally, for multitask knowledge transfer, the MTC module enables cross-task knowledge transfer through 2 auxiliary tasks: protein function prediction and protein subcellular localization prediction. This collaborative information across tasks improves the overall prediction level of protein interactions. These findings confirm that both the CCA and PCA modules are indispensable for DCAPPI’s predictive power, with the PCA module playing a more dominant role in PPI prediction. This emphasizes the biological importance of integrating partner-specific information in interaction modeling.

### Performance comparison

To fully verify the performance metrics of DCAPPI, we conduct extensive comparative experiments against 15 state-of-the-art PPI prediction methods across 3 benchmark datasets (the Yeast, Multi-species, and Multi-class datasets). These datasets are designed to evaluate different aspects of model capability: the Yeast dataset assesses performance on high-confidence single-species PPIs, the Multi-species dataset tests generalization under varying sequence identity constraints, and the Multi-class dataset examines the model’s ability to classify specific interaction types. All experiments retrain DCAPPI on the training set from combined folds and evaluate on the benchmark test set, which is the same as CollaPPI [11], with consistent evaluation metrics for fair comparison.

#### Comparison on the Yeast dataset

The Yeast dataset is a widely used benchmark for PPI prediction, consisting of 5,594 experimentally verified positive PPI pairs and 5,594 noninteracting negative pairs derived from 2,497 yeast proteins. This dataset is characterized by high-quality annotations, making it ideal for evaluating the core predictive power of models. Table 4 presents the performance comparison between DCAPPI and 15 competing methods, including traditional ML approaches and DL-based models. As shown in Table 4, DCAPPI outperforms all competing methods across key metrics (ACC, PRE, F1, and MCC), which are critical for assessing PPI prediction accuracy.

**Table 4.** Performance comparison with current methods on the Yeast dataset. Notes: NA means not available. The best results are shown in bold.

Methods	ACC (%)	PRE (%)	SEN (%)	SPE (%)	F1 (%)	MCC (%)
kNN–CTD	86.15	90.24	81.03	NA	85.39	NA
PCA–EELM	86.99	87.59	86.15	NA	86.86	77.36
SVM–MCD	91.36	91.94	90.67	NA	91.30	84.21
DeepPPI	94.43	96.65	92.06	NA	94.30	88.97
RF–LPQ	93.92	96.45	91.10	NA	93.70	88.56
SAE	67.17	66.9	68.06	66.3	67.44	34.39
DPPI	94.55	96.68	92.24	NA	94.41	NA
DNN-PPI	76.61	75.106	79.63	73.59	77.29	53.32
PIPR	97.09	97.00	97.17	97.00	97.093	94.17
MARPPI	96.03	98.128	93.51	**98.82**	NA	91.83
DAEPPI	97.85	97.071	98.70	NA	NA	95.73
KSGPPI	97.64	97.44	97.85	97.43	97.62	95.30
TAGPPI	97.81	98.10	98.26	98.10	97.80	95.63
CollaPPI	97.77	97.76	97.76	97.77	97.76	95.53
PPI-BAN	98.28	98.15	**98.41**	98.15	98.28	96.56
DCAPPI	**98.57**	**98.74**	98.39	98.74	**98.57**	**97.14**

ACC, accuracy; PRE, precision; SEN, sensitivity; SPE, specificity; F1, F1-score; MCC, Matthews correlation coefficient; kNN, *k*-nearest neighbors; SVM, support vector machine; RF, random forest; DCAPPI, Dual Cross-Attention network for Protein-Protein Interaction prediction

In terms of F1-score and MCC, which are robust to class distribution and sensitive to prediction reliability, DCAPPI achieves values of 98.57% and 97.14%, respectively, outperforming the second best method PPI-BAN [7] by 0.29% and 1.49%. This improvement highlights DCAPPI’s superior ability to balance precision and recall, minimizing both false positives and false negatives. For ACC and PRE, DCAPPI reaches 98.57% and 98.74%, respectively, which are 0.29% and 0.59% higher than those of PPI-BAN [7]. This demonstrates DCAPPI’s robustness in correctly classifying interacting and noninteracting pairs, as well as its high confidence in positive predictions. While DCAPPI’s SEN (98.39%) and SPE (98.74%) are slightly lower (by 0.31% and 0.08%) than those of DAEPPI and MARPPI, this trade-off is justified by its overall superiority across more comprehensive metrics (F1 and MCC). Unlike DAEPPI and MARPPI, DCAPPI achieves a better balance across all dimensions, which is an essential property for practical PPI prediction.

The results observed from Table 4 confirm that the dual cross-attention mechanism (CCA and PCA) in DCAPPI effectively captures intra-protein sequence and structure dependencies, and inter-protein interaction patterns. This way of feature extraction and fusion outperforms traditional methods that rely on handcrafted features or shallow fusion strategies, as well as DL models that treat sequence/structure or protein pairs as isolated entities.

#### Comparison on the Multi-species dataset

**Fig. 6.**
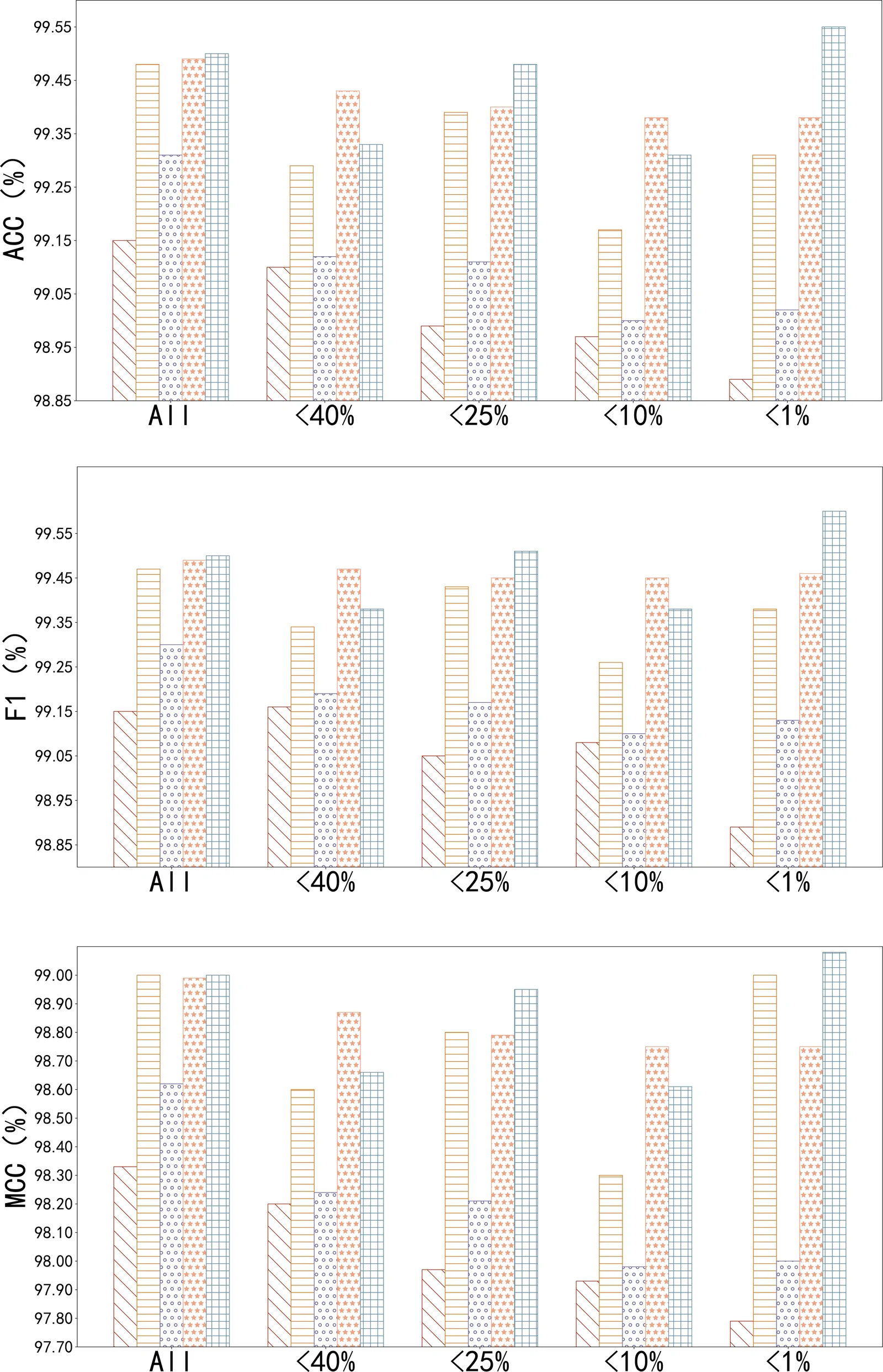
Performance comparison with current methods on the Multi-species dataset. Subplots show the accuracy (ACC), Matthews correlation coefficient (MCC), and F1-score of DCAPPI and state-of-the-art methods across the All and <40%, <25%, <10%, and <1% sequence identity subsets.

The Multi-species dataset integrates proteins from 3 distinct organisms (*E. coli*, *C. elegans*, and *D. melanogaster*) and is partitioned into subsets based on sequence identity thresholds (1%, 10%, 25%, 40%, and the All subset). This design allows us to evaluate model generalization under low-sequence-identity conditions, which is a critical challenge in PPI prediction, as many functionally relevant PPIs involve proteins with limited sequence similarity. Figure 6 presents the performance comparison between DCAPPI and state-of-the-art methods across these subsets.

The results shown in Fig. 6 reveal that DCAPPI exhibits exceptional generalization ability. Under the most stringent condition (1% sequence identity), DCAPPI achieves the best performance among all methods, with an ACC of 99.55% and an MCC of 99.08%. This outperforms CollaPPI (the second best method) by 0.53% in ACC and 1.08% in MCC, demonstrating DCAPPI’s ability to capture evolutionarily conserved structural and functional cues that are independent of sequence similarity. This advantage under low sequence identity mainly stems from 2 aspects. First, the ESM pre-trained PLM provides generalizable residue representations that capture conserved biochemical properties and local structural propensities across evolutionarily distant proteins, which forms a reliable basis for cross-homology prediction. Second, the contact-map-based structural topology encoding captures folding and binding patterns that are far more conserved than primary sequences, which further reduces the model’s dependence on sequence similarity. For subsets with higher sequence identity (10% and 40% sequence identity), DCAPPI’s performance is second only to that of PPI-BAN, with ACC values exceeding 99%. Moreover, DCAPPI achieves the best performance on all metrics with the 25% sequence identity subset and the All dataset. This indicates that DCAPPI can effectively leverage sequence similarity when available while maintaining robustness when sequence information is limited. These findings highlight DCAPPI’s practical utility for large-scale PPI prediction across diverse species, where many proteins lack close homologs with known interactions.

#### Comparison on the Multi-class dataset

**Fig. 7.**
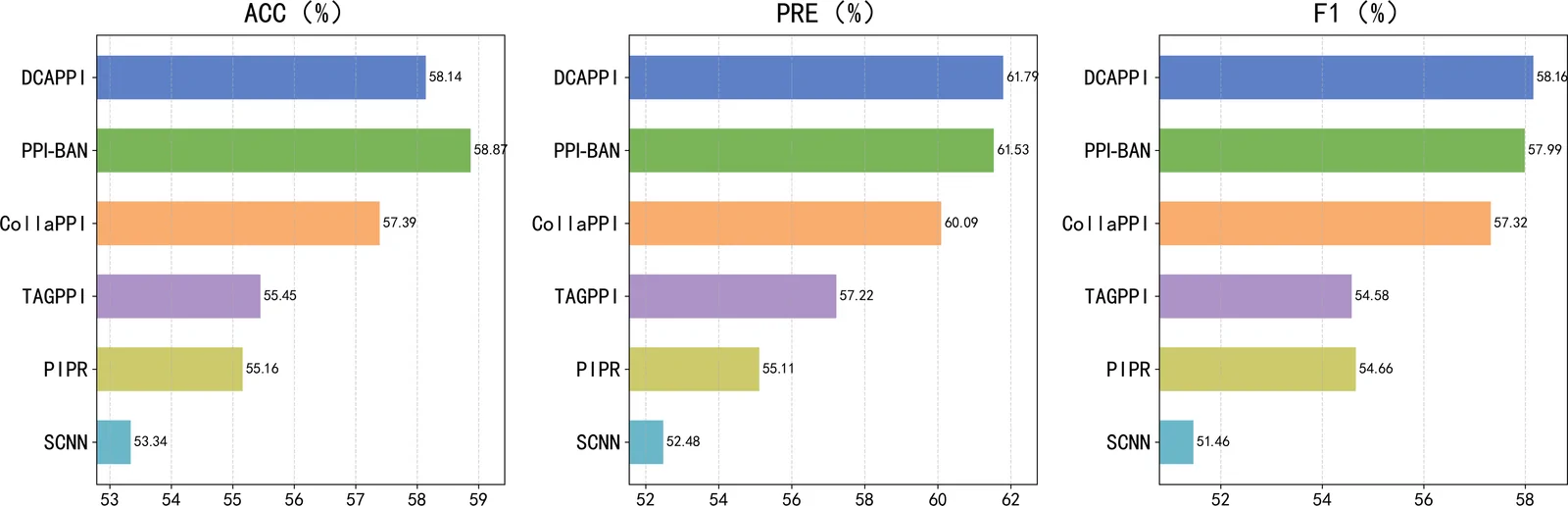
Performance comparison on the Multi-class dataset.

Unlike the binary classification task of predicting whether 2 proteins interact on the Yeast and the Multi-species datasets, the Multi-class dataset requires models to classify 7 distinct PPI types, which are binding, catalysis, reaction, posttranslational modification, inhibition, expression, and activation. This dataset evaluates a PPI prediction model’s ability to capture context-specific interaction mechanisms, which is a more complex and biologically meaningful task because different interaction types underpin distinct cellular processes. Figure 7 presents the performance of DCAPPI and competing methods on this dataset.

As illustrated in Fig. 7, DCAPPI demonstrates strong robustness and discriminative power for multiclass PPI classification. In terms of ACC, DCAPPI achieves 58.14%, second only to that of PPI-BAN [7] (58.87%), with a marginal gap of 0.73%. For PRE (61.79%) and F1-score (58.16%), DCAPPI outperforms all competing methods, including the second best PPI-BAN [7], with improvements of 0.26% and 0.17%, respectively. This indicates that DCAPPI not only correctly identifies interaction types but also maintains high confidence in its predictions. This is a critical feature for applications such as drug target identification, where precise classification of interaction mechanisms can guide therapeutic design.

#### Comparison on the gold standard dataset

To rigorously evaluate the true generalization capability of DCAPPI and rule out performance inflation caused by dataset biases [63], we conduct supplementary validation on the gold standard dataset [62]. This dataset is specifically constructed to eliminate sequence similarity leakage and node-degree biases that are pervasive in traditional PPI benchmarks, and it has been shown that most existing methods perform near randomly on this benchmark.

We strictly follow the official train/val/test split protocol about the gold standard dataset, adopt the optimal hyperparameter configuration determined by our ablation experiments (see the “Impact of the number of attention heads” and “Ablation studies” sections), and retrain the full DCAPPI model end to end on the training split with no exposure to test samples. The results are presented in Table 5.

**Table 5.** Performance comparison on the gold standard dataset. Notes: Values for TUnA [64] and C3PI [65] are taken from their original publications. The superscript “a” indicates that the metrics are reported by Bernett *et al.* [62]. The best results are shown in bold.

Model	SEN	SPE	PRE	ACC	F1	MCC	AUROC	AUPRC
D-SCRIPT^a^	0.194	0.813	0.509	0.503	0.280	0.009	0.499	0.510
DeepFE^a^	0.466	0.570	0.520	0.518	0.492	0.037	0.521	0.513
PIPR^a^	0.464	0.580	0.525	0.522	0.492	0.044	0.532	0.528
R-FC^a^	0.405	0.644	0.533	0.524	0.460	0.050	0.524	0.514
R-LSTM^a^	0.607	0.423	0.514	0.515	0.556	0.030	0.515	0.509
Topsy-Turvy^a^	0.261	**0.857**	0.646	0.559	0.372	0.146	0.587	0.590
C3PI	0.705	0.587	0.630	0.646	0.665	0.293	**0.703**	0.695
TUnA	0.650	0.650	**0.650**	**0.650**	0.650	**0.300**	0.700	0.690
DCAPPI	**0.726**	0.561	0.623	0.643	**0.671**	0.291	0.702	**0.743**

SEN, sensitivity; SPE, specificity; PRE, precision; ACC, accuracy; F1, F1-score; MCC, Matthews correlation coefficient; AUROC, area under the receiver operating characteristic curve; AUPRC, area under the precision–recall curve; DCAPPI, Dual Cross-Attention network for Protein-Protein Interaction prediction

It can be seen that most conventional PPI methods exhibit near-random performance on this strict benchmark. Compared with representative state-of-the-art methods, our DCAPPI model delivers competitive and superior overall performance. Specifically, DCAPPI achieves an area under the precision–recall curve of 0.743, an F1-score of 0.671, and a sensitivity (SEN) of 0.726 performing best and maintains a comparable area under the receiver operating characteristic curve of 0.702. These results empirically demonstrate that the performance advantage of DCAPPI does not stem from dataset biases or information leakage and that the model captures genuine interaction patterns underlying protein binding.

#### Case study

**Fig. 8.**
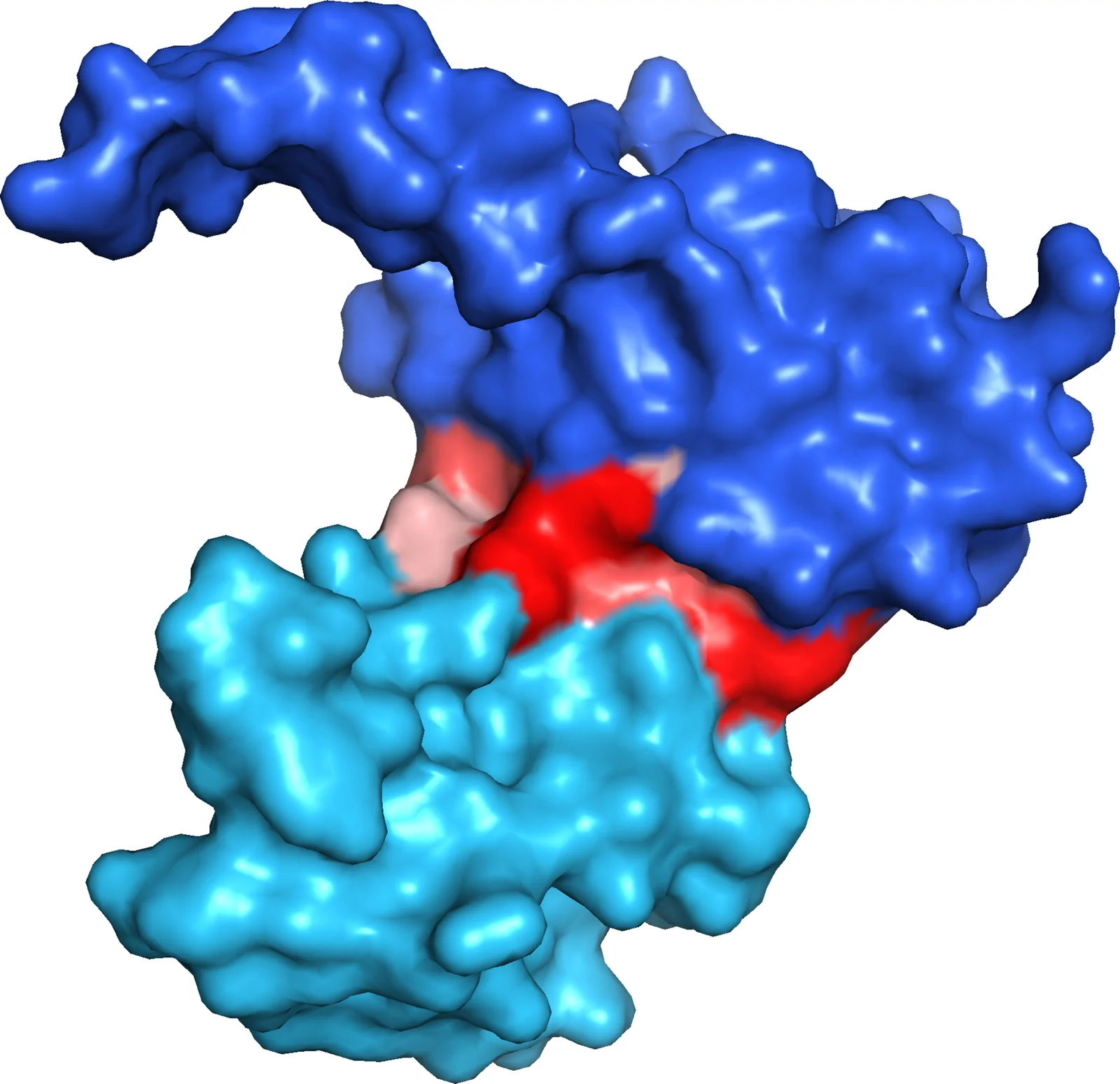
Attention weight visualization mapped onto the 3D molecular surface of the human protein complex (Protein Data Bank [PDB] ID: 3BS5). The 2 interacting polypeptide chains are rendered in dark blue and cyan, respectively. Residues with the highest attention scores derived from the Partner Cross-Attention (PCA) module are highlighted in red.

To verify that DCAPPI captures biologically meaningful interaction patterns, we perform a structural case study using the experimentally solved human protein complex (Protein Data Bank ID: 3BS5) as an independent test case. We feed the 2 chains of the 3BS5 complex into the DCAPPI model trained on the Yeast dataset, extract the residue-level mutual attention scores, and map them onto the 3D molecular surface of the complex for visualization. As shown in Fig. 8, the 2 interacting chains are rendered in dark blue and cyan, and residues with the highest attention scores are marked in red. High-attention residues are strongly concentrated at the physical binding interface, consistent with the biochemically verified interaction region.

## Discussion and Conclusion

PPIs underpin core cellular processes such as signal transduction, metabolic regulation, and disease pathogenesis, and accurate PPI prediction is critical for deciphering molecular mechanisms and identifying therapeutic targets. While DL has advanced computational PPI prediction, growing evidence indicates that many existing models overestimate their performance by exploiting dataset biases such as sequence homology leakage and node-degree shortcuts, limiting their utility in real-world biological screening. In this work, we present DCAPPI, a dual cross-attention framework that performs hierarchical feature fusion at both intra-protein and inter-protein levels. Below, we discuss the mechanistic insights, generalization capability, biological interpretability, and limitations of the proposed method and conclude with its implications and future directions.

The core advantage of DCAPPI lies in its 2-level cross-attention fusion paradigm. At the intra-protein level, the CCA module enables bidirectional information flow between BiGRU-captured sequential context and GAT-encoded structural topology. Ablation results confirm that cross-attention fusion outperforms naive concatenation by dynamically weighting functionally relevant residues across modalities. Although both branches are initialized from ESM-2 embeddings, contact map topology provides independent spatial constraints that cannot be replaced by sequence features alone. At the inter-protein level, the PCA module models interacting protein pairs as coupled units instead of isolated entities. Removing PCA causes the most severe performance drop, verifying that modeling inter-protein contextual dependencies is critical for distinguishing specific interactions from random pairings.

To address widespread dataset bias in PPI evaluation, we systematically evaluate DCAPPI under multiple rigorous settings, including the Yeast, Multi-species, Multi-class, and gold standard datasets. DCAPPI maintains stable performance across all these datasets among compared methods. This robustness mainly stems from the integration of predicted 3D structural features, which are more evolutionarily conserved than primary sequences and effectively alleviate the shortcut learning problem prevalent in sequence-only models. Attention visualization on experimentally protein complexes further supports the biological rationality of our design, indicating that DCAPPI captures biologically meaningful interaction patterns rather than statistical artifacts in training datasets.

Despite the improved performance, DCAPPI still has several limitations. The model depends on predicted protein structures as input, and while a ColabFold-based fallback pipeline is available for proteins without AlphaFold DB entries, its performance may degrade for intrinsically disordered proteins or low-quality structure inputs. In addition, the current structural branch only utilizes $C_{\alpha }$ contact maps for graph construction, without explicit modeling of fine-grained 3D geometric features such as residue orientation and dihedral angles, which restricts the structural modality’s ability to capture detailed biophysical interactions. Moreover, the main benchmarks still adopt randomly constructed negative samples, and the model’s generalization in ultralarge-scale *de novo* interactome screening needs further validation. Finally, the auxiliary tasks in the MTC module cover only high-frequency GO terms and localization annotations, with limited coverage of protein functional diversity.

In conclusion, DCAPPI provides a robust hierarchical cross-attention fusion paradigm for PPI prediction, which effectively addresses the oversimplified feature fusion strategies and isolated feature processing limitations in existing methods. By integrating AlphaFold-predicted 3D structures and ESM-2 sequence embeddings, DCAPPI achieves state-of-the-art performance on 3 conventional benchmarks and maintains strong generalization under low sequence identity and unbiased evaluation settings. The MTC module further enhances the biological relevance of predictions by incorporating functional and subcellular localization knowledge. Building on this work, future research will focus on integrating distance-aware GNNs and equivariant operations to enrich 3D geometric modeling, designing hard-negative-aware training strategies to improve real-world screening performance, developing a user-friendly web server with attention visualization and binding site annotation, and extending the framework to multiprotein complex and transient interaction prediction. We hope that DCAPPI can serve as a reliable tool to accelerate biological research, guide experimental design, and advance our understanding of complex cellular interaction networks.

## Data Availability

Data and codes from this study are available at https://github.com/biolushuai/Dual-Cross-Attention-Network-for-PPI-Prediction.
